# The FAR protein family of parasitic nematodes

**DOI:** 10.1371/journal.ppat.1010424

**Published:** 2022-04-21

**Authors:** Sophia C. Parks, Susan Nguyen, Martin J. Boulanger, Adler R. Dillman

**Affiliations:** 1 Department of Nematology, University of California, Riverside, California, United States of America; 2 Department of Biochemistry and Microbiology, University of Victoria, Victoria, Canada; University of Pennsylvania, UNITED STATES

## Abstract

Fatty acid–and retinol-binding proteins (FARs) belong to a unique family of excreted/secreted proteins (ESPs) found exclusively in nematodes. Much of our understanding of these proteins, however, is limited to their in vitro binding characteristics toward various fatty acids and retinol and has provided little insight into their in vivo functions or mechanisms. Recent research, however, has shown that FARs elicit an immunomodulatory role in plant and animal model systems, likely by sequestering lipids involved in immune signaling. This alludes to the intricate relationship between parasitic nematode effectors and their hosts.

## Significance

Parasitic nematodes infect billions of people worldwide as well as other mammals, insects, and plants [1,2]. The soil-transmitted helminth *Ascaris lumbricoides* alone infects approximately 1 billion people globally, while plant-parasitic nematodes cause billions of dollars in crop damage every year [3]. During infection, nematodes release a complex mixture of excreted/secreted proteins (ESPs) into surrounding host tissues that can interfere with host signaling mechanisms and immune homeostasis, allowing for a more successful infection [4–6]. Most of these ESPs have not been studied in detail, although such studies are essential for our understanding of host–helminth interactions as well as the outcome of infection. One unique protein family in nematode ESPs is the family of fatty acid–and retinol-binding proteins (FARs) [7]. Numerous studies describing the in vitro binding characteristics of FARs reveal that these proteins bind fatty acid and retinol molecules, including those important to immune signaling, and therefore have the potential to play a crucial role in modulating the host immune system. Despite the wealth of information describing the binding properties of FARs, however, little is known about how these proteins interact with host tissues and potentially alter host immunity [8–20].

## Introduction and history of nematode lipid binding proteins

Nematodes are unable to synthesize all necessary lipids de novo and have therefore evolved sophisticated, protein-based mechanisms to sequester lipids and related precursor molecules from diet and the environment [13]. Underpinning key aspects of nematode biology are the nematode polyprotein antigens (NPAs), venom allergen–like proteins (VALs), and FARs [21]. NPAs are small, helix-rich proteins that are initially synthesized as a large polyprotein before being cleaved into functional copies of approximately 15 kDa in size [4,12,14,22–24]. NPAs bind to fatty acids and retinoids and are secreted by nematodes and can elicit a strong host immune response [12,22,25]. VALs are up-regulated during the parasitic life stage and can bind lipids. VALs have also been studied for vaccine development and their roles in host–parasite interactions [26].

Among fatty acid–binding proteins, FARs are an understudied and uniquely nematode protein family having no orthologs in other animals or plants [27]. Initially, FARs attracted attention for their ability to bind retinoids and therefore play a role in parasitic growth, differentiation, and reproduction [28]. The initial significance of this retinoid binding role was analyzed primarily in the context of the nematode’s own biological functions. In *Onchocerca volvulus* infections, for example, the concentration of retinol was shown to be much greater in the nematode than in the surrounding host tissue where it was hypothesized to play a role in growth and reproduction [29]. Moreover, retinoic acid, the primary metabolite derived from retinol, is localized to developing embryos and is required for normal growth and development [28]. Further studies revealed that FAR proteins can bind fatty acids in addition to retinol [11,30]. More recently, the presence of FARs in ESPs and their ability to bind host lipids support a potential role in parasitism through immunomodulation that could be leveraged for vaccine development [11,24,25].

FARs also have distinct features that allow for their secretion into and interaction with surrounding host tissues. For example, the presence of a casein kinase II phosphorylation site in addition to a hydrophobic leader signal peptide is thought to play a key role in regulation of FARs’ secretion into host tissue [8,10,11,15,17,31]. Metabolic labeling experiments revealed the presence of FAR proteins in supernatant of adult parasite cultures of different species, which confirms the presence of FAR proteins in the excretory/secretory products of filarial parasites [32]. Collectively, FARs are an important family of proteins that garner significant interest for their roles in immune modulation, parasitism, and as potential therapeutic targets.

### Diversity of FARs

FAR proteins contain on average 130 to 170 amino acids [8,10,11,17,33], and the number of FARs encoded in each nematode genome appears to be lineage specific. For example, *Brugia malayi* contains 3 FAR proteins, *Caenorhabditis elegans* contains 9, and the insect parasitic nematode *Steinernema carpocapsae* contains 45 putative FAR proteins, leading to the hypothesis that FAR proteins play a significant role in parasitism and are expanded to ensure interaction with host tissues [11,16,18,19,34–36]. For a comprehensive evolutionary analysis of FAR proteins, readers are referred to a recent study by Yuan and colleagues [27]. Notably, when multiple FAR proteins exist in a species, they tend to have distinct sequences and display biochemical and functional variability. For example, both *Heterodera avenae* Ha-FAR-2 and *B*. *malayi* Bm-FAR-2 have been reported to have weaker binding affinities to retinol and fatty acids compared to Ha-FAR-1 and Bm-FAR-1, respectively [19,36].

The diversity of FARs is also observed at the level of posttranslational modifications such as glycosylation. For example, *O*. *volvulus* Ov-FAR-1 has 3 predicted glycosylation sites, *B*. *malayi* Bm-FAR-1 and *Loa loa* Ll-FAR-1 have 1, while *Brugia pahangi* Bp-FAR-1and *Wuchereria bancrofti* Wb-FAR-1 have none [37]. Glycosylation in FARs is *N-*linked, although it has been found that the location for glycosylation can differ between species—Bm-FAR-1 and Ll-FAR-1 glycosylation sites, for example, are distinct from the 3 sites of Ov-FAR-1. Degree of glycosylation can also vary, as seen in *Onchocera* species where FAR proteins are differentially glycosylated, leading to native proteins with 2 different masses. In contrast, a FAR from *Acanthocheilonema viteae*, a filarial parasite of rodents, is predicted to have at least 1 glycosylation site, and only 1 native protein size is observed [37]. Although the specific function of glycosylation in FARs is still unclear, it has been noted that species with glycosylated FARs possess unsheathed microfilariae instead of sheathed, which might be linked to the transfer of FARs through the sheath [32].

**Fig 1:**
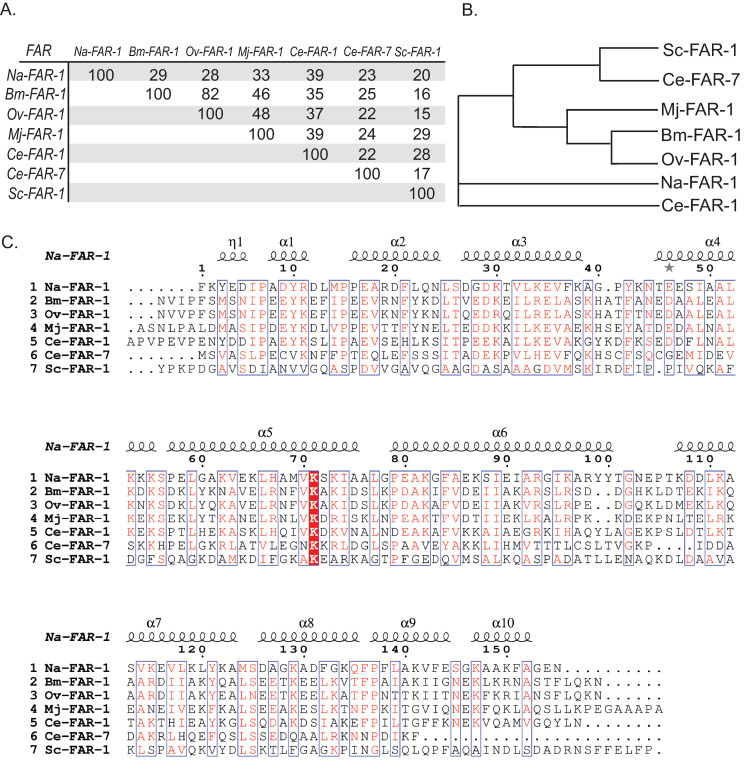
Comparison of FAR proteins across free-living and parasitic nematode species. **(A)** Table of percent identity among various FAR proteins showing Ov-FAR-1 with the highest similarity to Bm-FAR-1 (82 percent). **(B)** Phylogenetic tree of FAR sequences included in alignment created in ClustalW. **(C)** The FAR protein sequences are aligned with clustalW2 with secondary structures shown as an estimation based on the *N*. *americanus Na*-FAR-1 (4XCP) crystal structure using ESPript [23,51]. Sequence identities are as follows: Na-FAR-1 (XP_013293708), Bm-FAR-1 (Q93142), Ov-FAR-1 (Q25619), Mj-FAR-1 (AFZ77091), Ce-FAR-1 (CAA79616), Ce-FAR-7 (NP_493708), and Sc-FAR-1 (TKR66991). The various secondary structures are labeled α with squiggles for large alpha helices and η with squiggles for 3_10_ helices. Alternate residues are highlighted with gray stars. Residues that are identical among all groups are highlighted in red, and conserved residues are shown in red; both are outlined in blue. FAR, fatty acid–and retinol-binding protein.

Differing localization patterns in the nematode’s body also contributes to FAR protein diversity. Most FAR proteins in parasitic nematodes can be found in the hypodermis, cuticle surface region, and esophageal glands, which suggests their presence in nematode secretions and potential role in mediating host–parasite interactions [11,16,38]. FARs are also often found in reproductive glands, in larvae, and in higher amounts in females, suggesting a biological importance in development and reproduction in addition to secretion [11,13,23,39,40]. Moreover, sequence analysis of some of the most well-studied FARs reveals a wide-ranging degree of conservation at the protein level (Fig 1). Not surprisingly, FARs from closely related species, such as within the *Meloidogyne* clade, have higher a higher degree of sequence identity relative to those from more disparate clades [36,41].

### FARs adopt a unique alpha-helical bundle fold

**Fig 2:**
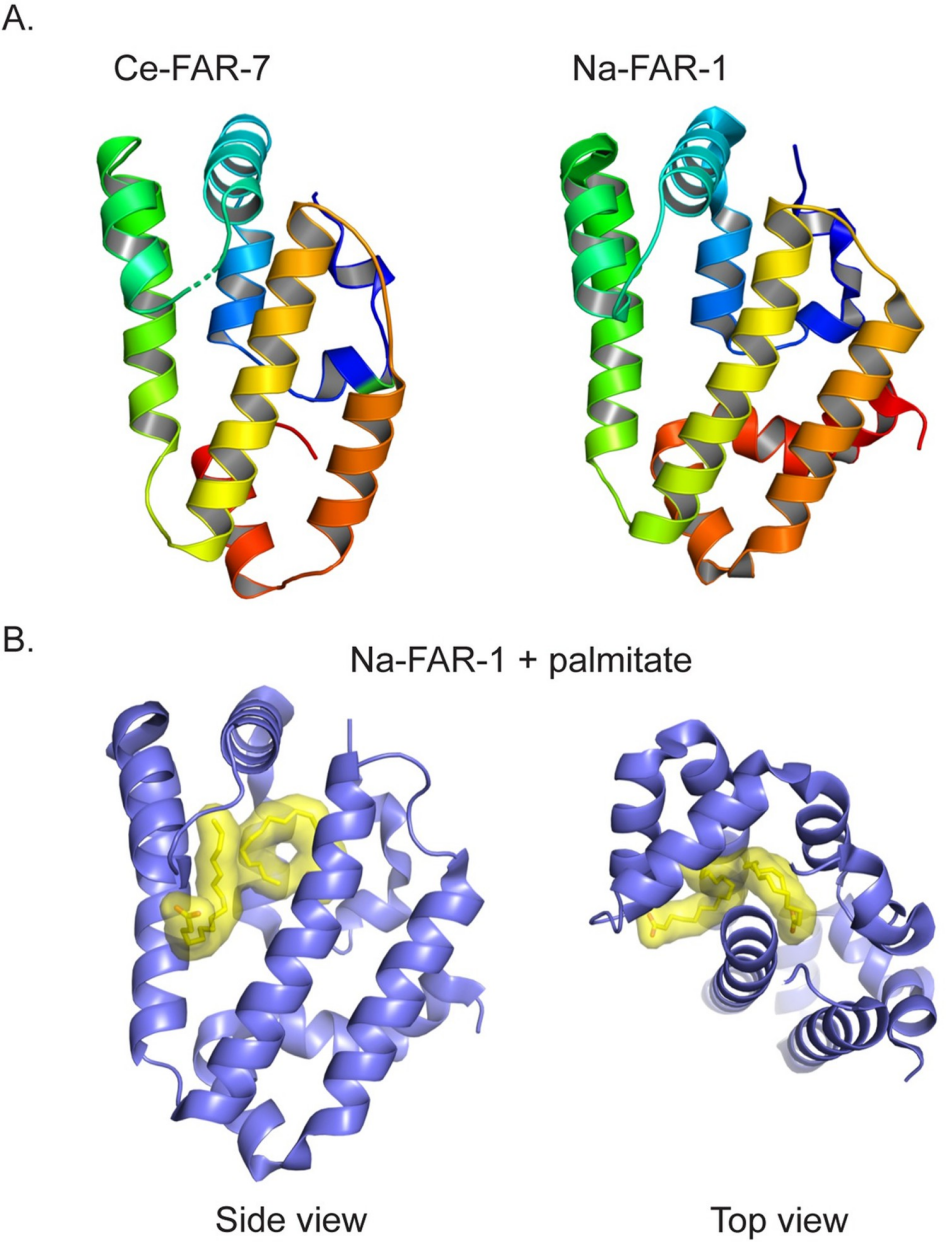
Secondary structure representations of the fatty acid and retinol binding proteins Ce-FAR-7 from *C*. *elegans* and Na-FAR-1 from *N*. *americanus*. **(A)** Rainbow color scheme of Ce-FAR-7 (2W9Y) and Na-FAR-1 (4XCP) ranging from blue (amino terminus) to red (carboxyl terminus) that highlights the conserved alpha-helical fold. **(B)** Side and top views of NA-FAR-1 bound to palmitate (yellow surface) that highlights the large central ligand binding cavity. Structure figures were generated using PyMOL (www.PyMOL.org). FAR, fatty acid–and retinol-binding protein.

Despite an abundance of available FAR sequence and biochemical information, only 2 FAR protein structures have been determined to date; the apo (ligand-free) structure of *C*. *elegans* Ce-FAR-7 was reported by Jordanova and colleagues in 2009 [31], and the apo and palmitate bound forms of *Necator americanus* Na-FAR-1 were reported by Rey-Burusco and colleagues in 2015 [23]. The original structural analysis of Ce-FAR-7 revealed a multihelix bundle architecture (Fig 2A, left panel) distinct from any previously defined lipid binding protein [31]. Ce-FAR-7, however, is notably phylogenetically distant from parasitic nematode FARs [32], such as Na-FAR-1, with which it shares approximately 23% sequence identity, lacks a signal peptide, and displays distinct lipid binding characteristics [32]. Despite these phylogenetic and functional differences, the overall helical architecture of Ce-FAR-7 is surprisingly well conserved in Na-FAR-1 (Fig 2A, right panel). Nuanced structural differences include the number of helices in the bundle, and the relative orientation and packing of the helices that, while not significantly altering the overall fold, are likely to influence ligand binding.

Several studies have shown that nematode FARs bind fatty acids and retinol [8,9,13,15,32], and the Ce-Far-7 and Na-FAR-1 structures provided important insight into the mechanism of ligand coordination. The apo Ce-FAR-7 structure revealed 2 deep hydrophobic pockets (P1 and P2) with the smaller P1 pocket predicted to bind fatty acids and the larger P2 pocket predicted to bind bulkier retinoids [31]. The P1 and P2 pockets, which are joined by a cleft that authors postulated, could facilitate binding of multiple different ligands of varying aliphatic chain length [31]. This hypothesis was ultimately confirmed through steady-state fluorescence spectroscopy titration experiments that showed binding of chemically and structurally different ligands, some of which were able to displace retinol bound in the P2 pocket [31]. The successful determination of the apo and palmitate bound Na-FAR-1 structures allowed for a more detailed mapping of the internal hydrophobic cavities (Fig 2B, palmitate ligands shown as yellow surfaces) [23] including characterizing the structural changes associated with ligand binding. Structural analysis also revealed the potential for more than 1 entry point for hydrophobic molecules to access the internal cavities facilitated by mobile alpha helices that may serve as structural gatekeepers. Fluorescence-based binding studies with Na-FAR-1 showed binding of the fluorescent fatty acid analogue 11-(Dansylamino) undecanoic acid (DAUDA) and the naturally fluorescent lipid retinol [23]. Intriguingly, Ce-FAR-7 does not appear to bind DAUDA [32], and binding to retinol showed no saturation [31]. The Na-FAR-1 study also expanded the repertoire of potential ligands to include a broad range of lipid classes including phospholipids. The diversity in hydrophobic ligands bound by FARs may provide parasitic nematodes the ability to colonize a variety of biological niches. Further studies, however, are required to correlate in vitro binding studies with biological outcomes.

### Plant-parasitic nematode FAR functions

Much work has been done to evaluate the roles of FARs in plant parasite interactions and has served as a foundation to understand the immunomodulatory effects of these unique proteins. Plant-parasitic nematode FARs have been shown to positively affect the nematode infection process. For example, the first plant FAR discovered was Gp-FAR-1 in *Globodera pallida* that binds to precursors of the jasmonic acid signaling pathway and inhibits lipoxygenase activity in vitro [8]. Lipoxygenase activity is part of the octadecanoid signaling pathway that eventually leads to the synthesis of jasmonic acid, a signal transducer in systemic plant immunity. Jasmonic acid mediates responses against environmental stress, which can range from herbivore damage to pathogen infection by inducing expression of genes that produce chemical defense compounds such as alkaloids and terpenoids [42]. By interfering with this pathway, Gp-FAR-1 likely modulates host immunity and thereby contributes to a more successful nematode infection. *Bursaphelenchus xylophilus* Bx-FAR-1 expression levels are up-regulated in the earlier infection stages of *B*. *xylophilus*, and RNA interference (RNAi) of this FAR reduced infection rates [40]. The expressions of *pr-6* and *lox-5*, genes that are part of the jasmonic acid immune response pathway, are also found to be much higher when Bx-FAR-1 is silenced, suggesting that Bx-FAR-1 could interfere with this immune response process upon infection [40]. In *Meloidogyne incognita*, knockdown experiments of *Mi-far-1* also showed that the infection process and parasite reproduction are greatly reduced when Mi-FAR-1 is reduced. Mi-FAR-1 also appears to play a role in nematode defense against bacterial infection, as its reduction results in increased bacterial endospore attachment [43]. In *Pratylenchus penetrans*, suppression of Pp-FAR-1 protein reduced nematode reproduction by up to 70% compared to control lines [33].

The strongest evidence that FARs alter susceptibility to infection in plants is associated with *Melodogyne javanica* Mj-FAR-1 that influences parasitic infection of tomato roots. Transgenic tomato roots constitutively expressing Mj-FAR-1 showed a higher susceptibility to nematode infection and allow for faster nematode growth once infected. RNA interference experiments showed that nematode maturation slows when *Mj-far-1* is silenced [44]. Furthermore, expression of *Mj-far-1* resulted in suppression of jasmonic acid responsive genes such as *pin2* and *Ɣ-thionin*, similar to findings in Bx-FAR-1 research, although LOX gene expression is not significantly affected [44]. These are striking data on phenotypic changes in immunity and resilience to infection in plants. Silencing of *Ha-far-1* resulted in a significant reduction in reproduction of *H*. *avenae*, and analysis of gene expressions showed that *Ha-far-1* transcript levels during parasitic stages are higher compared to nonparasitic stages [16,36].

In *Radopholus similis*, comparison between a highly pathogenic population (Rs-C) and a less pathogenic population (Rs-P) showed that *Rs-far-1* expression is 2.5 times higher in the highly pathogenic population. RNA interference assays also indicated that Rs-FAR-1 regulates levels of allene oxide synthase (AOS), a component of the jasmonic acid pathway, and a reduction in reproduction and pathogenicity was also observed after *Rs-far-1* knockdown, and *Rs-far-1* expression level is also increased in the more pathogenic nematode population [20]. In *Arabidopsis thaliana*, compared to control plants, AOS expression is significantly decreased when treated with regular *R*. *similis*, but is significantly increased when treated with *Rs-far-1*-silenced *R*. *similis* [20]. Taken together, these findings suggest that FAR proteins assist in parasitic infections by manipulating the host plant jasmonic acid immune signaling pathway and contribute to the nematodes’ reproduction in host tissues.

### Animal-parasitic nematode FAR functions

Research on the immunomodulatory effects of FARs in animal-parasitic nematodes is much more limited compared to their plant-parasitic counterparts. FARs are secreted into host tissues during parasitic nematode infection, yet little is known about how FARs interact with host tissues [45,46]. In *Strongyloides stercoralis*, analysis of differential gene expression showed that in the infective life stage, a gene coding for a FAR protein is among the most highly expressed genes, suggesting that the protein plays a significant role during the infection process [39]. Bm-FAR-1 and Bm-FAR-2 from *B*. *malayi* are targets of strong IgG1, IgG3, and IgE antibody responses in infected individuals, showing that FARs are targets of host immune responses during a nematode infection [19]. The stage-dependent abundance of FARs and their ability to elicit strong immune responses in mammalian hosts hint at their importance during infection processes.

In *S*. *carpocapsae*, experiments with FAR-expressing transgenic *Drosophila melanogaster* showed that FAR proteins directly modulate host immunity [47]. Flies expressing or injected with Sc-FARs exhibited a significant decrease in C18s and their oxylipin derivatives, such as linoleic acid, 9,(10)-EpOME, and 9-HODE, a decrease in resistance to bacterial infection, and a significant reduction in other aspects of fly immune responses such as the phenoloxidase cascade and antimicrobial peptide production [47]. Sc-FAR-1 and Sc-FAR-2 bound strongly to linoleic acid and 9,(10)-EpOME in vitro and altered availability of linoleic and oleic acids, 9,(10)- and 12,(13)-EpOME, 9,(10)- and 12,(13)-DiHOME as well as 9- and 13-HODE in circulation in vivo, suggesting a mechanism for immunomodulation in animals through depletion of lipid signaling molecules necessary for immune response pathways [47]. These C18s and oxylipin derivatives are hypothesized to act as signaling molecules in a similar way to eicosanoids function in mammalian systems [48]. Little is known about the immunomodulatory role of the retinol binding pocket; however, retinol and retinoic acid are known to play a role in the differentiation and maturation of innate immune cells [49]. Currently, the strongest evidence supporting an immunomodulatory role for FARs in an animal system are derived from an insect model [47], and so more experiments need to be done in a mammalian model system to elucidate the connection between FAR proteins and host immunity.

### FARs as a target for therapeutic designs

Parasitic nematode FAR proteins do not have an equivalent in mammals [27], highlighting the possibility that they may serve as a potential new target for antihelminthic treatments [29,50]. For example, ivermectin, one of the main drugs used to treat helminth infections, has been shown to inhibit the function of FARs by competing with ligands in the retinol-binding sites [50]. Moreover, FARs elicit a strong antibody response in animal hosts, highlighting their potential as vaccine candidates targeting parasitic nematodes. Intriguingly, early vaccination experiments have shown a decrease in worm burden in vaccinated hosts upon infection [13], and vaccination of gerbils with recombinant Bm-FAR-1 lead to a significant reduction in adult *B*. *malayi* worms, alluding to the importance of FARs to nematode survival in the host [19].

## Conclusions

FARs are a unique protein family found in most nematodes, but there are no obvious orthologs in plants or other animals. They have immunosuppressive effects on plant and animal host immune systems including the jasmonic acid pathway, phenoloxidase activity, and antimicrobial peptide production. Detailed ligand binding and structural studies combined with functional characterization support a role for FARs in modulating host immunity. Future work to further evaluate the role of FARs in the complex mammalian immune system will lead to a better understanding of immune signaling in helminth infections. Interdisciplinary work between plant and animal model systems will also aid to further elucidate FARs’ functions in parasitism.
